# Construction of efficient xylose utilizing *Pichia pastoris* for industrial enzyme production

**DOI:** 10.1186/s12934-015-0206-8

**Published:** 2015-02-21

**Authors:** Pengfei Li, Hongbing Sun, Zao Chen, Yin Li, Taicheng Zhu

**Affiliations:** CAS Key Laboratory of Microbial Physiological and Metabolic Engineering, Institute of Microbiology, Chinese Academy of Sciences, Beijing, 100101 China; National Engineering Laboratory for Industrial Enzymes, Tianjin Institute of Industrial Biotechnology, Chinese Academy of Sciences, Tianjin, 300308 China; Department of Chemical and Biochemical Engineering, College of Chemistry and Chemical Engineering, Xiamen University, Xiamen, 361005 China

**Keywords:** Xylose, Xylose isomerase, Recombinant *Pichia pastoris*, Evolutionary engineering, Industrial enzymes

## Abstract

**Background:**

Cellulosic biomass especially agricultural/wood residues can be utilized as feedstock to cost-effectively produce fuels, chemicals and bulk industrial enzymes, which demands xylose utilization from microbial cell factories. While previous works have made significant progress in improving microbial conversion of xylose into fuels and chemicals, no study has reported the engineering of efficient xylose utilizing protein expression systems for the purpose of producing industrial enzymes.

**Results:**

In this work, using *Pichia pastoris* as an example, we demonstrated the successful engineering of xylose metabolizing ability into of protein expression systems. A heterologous XI (xylose isomerase) pathway was introduced into *P. pastoris* GS115 by overexpressing the *Orpinomyces* spp. XI or/and the endogenous XK (xylulokinase) gene, and evolutionary engineering strategies were also applied. Results showed that the XI pathway could be functionally expressed in *P. pastoris*. After 50 generation of sequential batch cultivation, a set of domesticated recombinant *P. pastoris* strains with different performance metrics on xylose were obtained. One evolved strain showed the highest xylose assimilation ability, whose cell yield on xylose can even be comparable to that on glucose or glycerol. This strain also showed significantly increased β-mannanase production when cultured on xylose medium. Furthermore, transcription analysis of xylose pathway genes suggested that overexpression of XI and XK might be the key factors affecting effective xylose assimilation.

**Conclusions:**

To our best knowledge, this study is the first work demonstrating the construction of efficient xylose utilizing *P. pastoris* strains, thus providing a basis for using cellulosic biomass for bulk industrial enzyme production.

## Background

Growing energy crisis and environmental pressures led to renewed interest in cellulosic biomass as a renewable feedstock for the production of fuels and chemicals. In recent years, cellulosic biomass especially agricultural/wood residues were exploited to produce value added products such as ethanol, xylitol, citric acids, lactic acid and other organic acids [1]. In addition to fuels and chemicals, there is also growing interest in utilization of agro-waste for fermentation of bulk enzymes including xylanase [2], lipase [3], cellulase [4], amylase [5] etc., which are needed in large volumes, but have a relatively low unit value so that significantly lower manufacturing costs are demanded.

To date, one of the main problems impeding commercial conversion of cellulosic biomass into value added products is the inefficient microbial utilization of xylose [6,7], which is a major constituent of cellulosic biomass feedstock and the second most abundant carbohydrate in nature. This challenge can be addressed by engineering xylose metabolism in microbial cell factories with no or low xylose utilizing ability. Such work has been performed in a variety of industrial workhorse like *Escherichia coli* [8], *Bacillus subtilis* [9], *Clostridium beijerinckii* [10], *Hansenula polymorpha* [11], *Corynebacterium glutamicum* [12] with the aim of converting cellulosic biomass into biofuels and other useful chemicals. Most notably, in recent years, there have been an explosive of studies reporting construction of xylose utilizing *Saccharomyces cerevisiae* strains for cellulosic ethanol production [13-15]. Unfortunately, while previous works have made significant achievement in improving microbial conversion of xylose into chemicals, no study has reported the building of xylose metabolic pathway into protein expression system with the aim of cost-effectively producing industrial enzymes. Since protein production in enzyme producers is often growth-associated during protein expression phase [16,17], producing strains which can most efficiently assimilate xylose into biomass are required. This is different from microbial conversion of xylose to chemicals, which is usually non-growth-associated and requires the maximum possible yield of product from the constructed strains.

In this work, as a proof-of-concept, we outlined the introduction of xylose utilization pathway into the enzyme producer *Pichia pastoris. P. pastoris* is one of the most successful eukaryotic expression systems developed in the past decade [18]. It showed great potential in the expression of a highly diverse of proteins and is most potent in the expression of several bulk enzymes, like phytase [19], mannanase [20], cellulase [21] and lipase [22]. Previous literature seems to indicate that *P. pastoris* are not able to utilize xylose as sole carbon source [23]. In this work, we induced a heterologous xylose pathway into *P. pastoris* by overexpressing the xylose isomerase (XI) from an anaerobic rumen fungus *Orpinomyces* spp. Combined with evolutionary engineering strategy, we finally were able to obtain a recombinant *P. pastoris* strain which can efficiently assimilate xylose for β-mannanase expression. This study is the first work demonstrating the construction of efficient xylose-utilizing *P. pastoris* strains.

## Results and discussion

### *P. pastoris* strain GS115 can assimilate xylose at slow rate

Whether *P. pastoris* can utilize xylose as carbon source was rarely reported in previous literature. One study by Inan et al. concluded that *P. pastoris* are not able to utilize xylose as sole carbon source [23]. In this work, the ability of *P. pastoris* to assimilate xylose was investigated in the first place.

**Figure 1:**
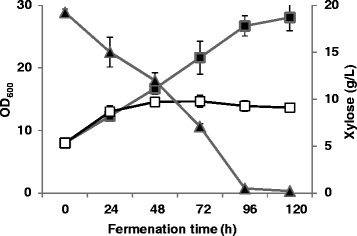
**Fermentation profiles of** ***Pichia pastoris*** **strain GS115 cultured in complex media with and without xylose.** Hollow square, cell growth without xylose; Solid square, cell growth with xylose; Solid triangle, residue xylose concentration. Three parallel flasks were tested.

The *P. pastoris* strain GS115 was inoculated into complex media with and without the addition of xylose. Results showed that without the presence of carbon source, the complex medium (which was rich in oligopeptides) can only sustain the growth of yeast cells for less than 48 h. Cell growth ceased at an OD_600_ of 14.5 and the cell mass began decreasing. In contrast, with the addition of xylose, cells kept growing for 120 h and reached a final OD_600_ of 28.1. Residue xylose concentration kept decreasing simultaneously and was depleted at 120 h. These results indicated that *P. pastoris* strain GS115 was able to assimilate xylose as a carbon source (Figure 1). However, the growth rate of GS115 on xylose was very low with a specific growth rate of 0.0075 h^−1^ (which approximates to a doubling time of 92 h; Table 1), which probably explains why xylose utilization has not been described for *P. pastoris*.

**Table 1:** **Plasmids and strains used in present study**

**Plasmids or strains**	**Relevant characteristics**	**Reference or source**
***Plasmids***		
pGAPZmazF	Vector for constitutive secreted protein expression; Zeo^r^	Invitrogen
pPIC9K	Vector for inducible secreted protein expression; HIS4; kan^r^ & Amp^r^	Invitrogen
pGAPZmazF (E,N)	pGAPZmazF based expression vector, containing *Eco*R I and *Not* I at multiple cloning site; Zeo^r^	Our lab
pGAPZH	pGAPZ based expression vector, carrying the HIS4 fragment from pPIC9K; Zeo^r^	Our lab
pGAPZ-XI-His	pGAPZ containing *XI* from *Orpinomyces* spp*.* and HIS4 fragment from pPIC9K; Zeo^r^	This study
pGAPZ-XK	pGAPZ containing *XK* from *P. pastoris* GS115; Zeo^r^	This study
pGAPKH-3Sman	pGAPKH containing three copies of alkaline β-mannanase gene, Kan^r^	[33]
***Strains***		
*E. coli* DH5α		Takara
*P. pastoris* GS115	his4^−^, Mut^+^	Invitrogen
*Orpinomyces* spp.		Our lab
GS-XI	GS115 integrated with the recombinant plasmid pGAPZ-XI-His	This study
GS-XK	GS115 integrated with the recombinant plasmid pGAPZ-XK	This study
GS-XI-XK	GS115 integrated with the recombinant plasmid pGAPZ-XI-His and pGAPZ-XK	This study
GS115^SB50^	GS115 with 50 generations of evolution	This study
GS-XI^SB50^	GS-XI with 50 generations of evolution	This study
GS-XK^SB50^	GS-XK with 50 generations of evolution	This study
GS-XI-XK^SB50^	GS-XI-XK with 50 generations of evolution	This study
GS-3Sman	GS115 integrated with the recombinant plasmid pGAPKH-3Sman	This study
GS-XI-3Sman	GS-XI^SB50^ integrated with the recombinant plasmid pGAPKH-3Sman	This study

**Figure 2:**
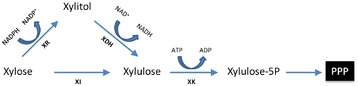
**Xylose utilizing pathway in engineered** ***Pichia pastoris*** **.** XR, putative xylose reductase (encoded by PAS_chr3_0744); XDH, putative xylitol dehydrogenase (encoded by PAS_chr1-1_0490); XI, xylose isomerase (derived from *Orpinomyces* spp.); XK, putative xylulokinase (encoded by PAS_chr1-1_0280). PPP, pentose phosphate pathway. The XR and XDH consist of the putative oxidoreductase xylose pathway of *P. pastoris*. The XI xylose pathway is heterologously introduced by over-expression of *Orpinomyces* spp. *XI* gene in *P. pastoris*.

In yeast species, xylose metabolism was started by its conversion to xylulose, which was mediated by two consecutive enzymatic reactions catalyzed by NADPH-dependent xylose reductase (XR) and NAD^+^-dependent xylitol dehydrogenase (XDH) (Figure 2). An examination over *P. pastoris* genome revealed an annotated gene (PAS_chr3_0744) encoding for putative XR and an unannotated gene (PAS_chr1-1_0490) homologous to XDH of other yeast species like *Saccharomyces cerevisiae* (56% identity), *Kluyveromyces marxianus* (57% identity), suggesting that *P. pastoris* genetically possesses the putative pathway for xylose assimilation. Further work such as genetic complementation tests are still required to validate this pathway.

### Introducing XI pathway alone did not significantly enhance xylose assimilation ability of *P. pastoris*

In order to enhance the xylose utilizing efficiency of *P. pastoirs*, we need to engineer an efficient xylose utilizing pathway in the host strain. In addition to the oxidoreductase (or XR/XDH) xylose pathway, another xylose pathway referred as XI pathway (which was found mainly in bacterial systems) can directly convert xylose to xylulose by xylose isomerase (XI) (Figure 2). The XI pathway is more desirable in xylose pathway engineering in recent years because it can eliminate the cofactor imbalance and the intermediate byproduct xylitol as normally observed in oxidoreductase xylose pathway. Therefore, in this work, we tried to enhance the flux from xylose towards pentose phosphate pathway (PPP) by introducing the XI pathway into *P. pastoris*.

**Figure 3:**
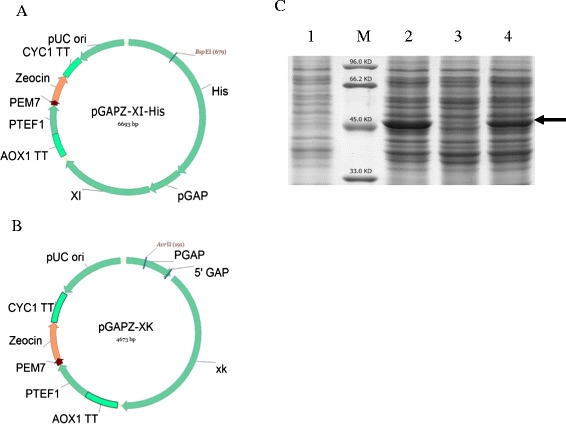
**Expression of XI and XK in** ***Pichia pastoris*** **. (A)** The expression vector containing the synthetic *XI* gene derived from *Orpinomyces* spp. **(B)** The expression vector containing the *P. pastoris XK* gene. **(C)** XI and XK expression analysis by SDS-PAGE. Lane 1, GS115 (control); Lane 2, GS-XI; Lane 3, GS-XK; Lane 4, GS-XI-XK; Lane M, protein molecular size marker. The right arrow indicate the position of XI.

The *XI* gene derived from *Orpinomyces* spp. was chosen because up to date only the *XI* from *Piromyces* and *Orpinomyces* [15] were functionally expressed in yeast system such as *S. cerevisiae*. In addition, previous work found that overexpression of xylulokinase (XK), which phosphorylates the xylulose to xylulose-5-phosphate (X5P) can also increase the flux towards the PPP [24]. Therefore, the *Orpinomyces* spp. *XI* gene (chemically synthesized, codon optimized) and endogenous putative *XK* gene (encoded by PAS_chr1-1_0280, amplified by PCR) were both placed under the strong constitutive GAPDH promoter of *P. pastoris*, resulting in the recombinant vectors pGAPZ-XI-His (Figure 3A) and pGAPZ-XK (Figure 3B). The two plasmids were separately or together transformed into GS115, thus generating three *P. pastoris* strains GS-XI, GS-XK and GS-XI-XK.

**Figure 4:**
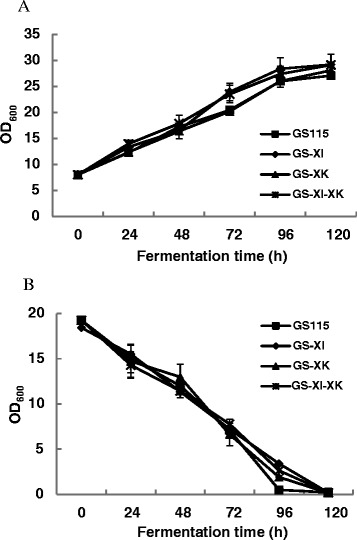
**Fermentation profiles of three engineered** ***P. pastoris*** **strains on xylose with GS115 as a control. (A)** Cell growth profile. **(B)** Xylose consumption profile. Three parallel flasks were tested for each stain.

SDS-PAGE analysis showed that XI could be successfully overexpressed in *P. pastoris*, as shown in GS-XI and GS-XI-XK (Figure 3C), which was then confirmed by MALDI-TOF MS analysis. The expression of XK cannot be detected in any strains by SDS-PAGE analysis perhaps due to its low expression level while can be verified at transcriptional level by qPCR analysis (data not shown). The four strains GS115 (control), GS-XI, GS-XK and GS-XI-XK were then tested for their xylose metabolizing ability using shake flask culture. Results showed that generally no significant difference in xylose fermentation profiles could be observed for GS-XI, GS-XK and GS-XI-XK as compared with GS115 (Figure 4A, B). Only a slight increase in cell growth over GS115 could be seen for GS-XI and GS-XI-XK at approximately 72 h of fermentation (Figure 4A). These results suggested that introducing the XI pathway may not be enough to ensure efficient xylose assimilation in *P. pastoris*. Metabolic adaption of yeast cells to xylose metabolism may be necessary, which can readily achieved by laboratory evolution.

### Xylose assimilation can be greatly improved by evolutionary engineering

**Figure 5:**
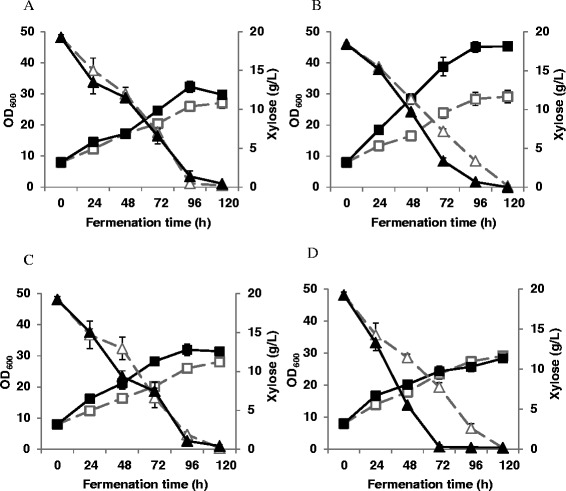
**Fermentation profiles of engineered** ***Pichia pastoris*** **strains with 50 generations of evolution. (A)** GS115^SB50^. **(B)** GS-XI^SB50^. **(C)** GS-XK^SB50^. **(D)** GS-XI-XK^SB50^. The growth profiles of respective parental strains are also presented for comparison. Square, cell growth profile; Triangle, xylose consumption profile; Dashed line, the parental strains; Solid line, the evolved strains. All experiments were performed in triplicate.

Evolutionary engineering strategy is widely applied to improve the pentose utilizing phenotype in yeast metabolic engineering [13,25,26]. The sequential batch cultivation method was used in this study with the aim of further improving xylose metabolizing ability of *P. pastoris*. After about 50 generations of evolution, we obtained evolved versions of GS115^SB50^, GS-XI^SB50^, GS-XK^SB50^ and GS-XI-XK^SB50^, respectively. The domesticated strains were then evaluated for their abilities to metabolize xylose by comparing their fermentation profiles (Figure 5) and calculated performance metrics (Table 2), which included specific growth rate (μ), specific substrate consumption rate (q_s_) and cell yield on xylose (Y_X/S_).

**Table 2:** **The calculated fermentation performance metrics for all engineered** ***Pichia pastoris*** **strains**

**Strains**	**μ (h** ^**−1**^ **)**	**q**_**s**_ **(g/g DCW/h)**	**Y**_**X/S**_ **(g/g DCW)**
GS115	0.0075 ± 0.0008	0.056 ± 0.006	0.135 ± 0.004
GS-XI	0.0107 ± 0.0014	0.055 ± 0.007	0.194 ± 0.007
GS-XK	0.0072 ± 0.0007	0.059 ± 0.008	0.123 ± 0.012
GS-XI-XK	0.0103 ± 0.0014	0.057 ± 0.008	0.181 ± 0.010
GS115^SB50^	0.0112 ± 0.0005	0.061 ± 0.006	0.185 ± 0.015
GS-XI^SB50^	0.0193 ± 0.0013	0.051 ± 0.004	0.378 ± 0.013
GS-XK^SB50^	0.0138 ± 0.0007	0.051 ± 0.006	0.271 ± 0.027
GS-XI-XK^SB50^	0.0110 ± 0.0013	0.092 ± 0.011	0.120 ± 0.003

μ, specific growth rate; q_s_, specific xylose consumption rate; Y_X/S_, cell yield on xylose. All metrics were average values calculated based on fermentation data at 72 h of fermentation. The contribution of oligopeptides present in the complex medium to performance metrics was excluded based on data from Figure 1.

Results showed that evolutionary engineering remarkably improved the xylose utilizing performance of all the four recombinant strains as expected, but in opposite manners. On one hand, as shown for GS115^SB50^, GS-XK^SB50^ and GS-XI^SB50^, the cell growth were significantly improved on xylose medium after 50 generation of evolution (Figure 5A, B, C). The average specific growth rates of GS115^SB50^, GS-XK^SB50^ and GS-XI^SB50^ were increased by 49%, 92% and 80%, respectively (Table 2). GS-XI^SB50^ reached an OD_600_ of 45.2 after 96 h of fermentation, which represented the highest value of all studied strains (Figure 5B). Nevertheless, the specific xylose consumption rates of GS115^SB50^, GS-XK^SB50^ and GS-XI^SB50^ were not improved or even slightly decreased (for GS-XI^SB50^ and GS-XK^SB50^), which led to increased cell yield on xylose for the three strains. Especially for GS-XI^SB50^, the Y_X/S_ on xylose reached a value of 0.378 g/g (Table 2), which can be comparable to that on glucose (0.310 g/g) [27] or glycerol (0.435 ~ 0.490 g/g) [27,28]. The results suggested that a significant amount of xylose flux was assimilated into the biomass of GS-XI^SB50^, and construction of XI pathway combined with evolution engineering could be a powerful strategy to improve the xylose assimilation capability of *P. pastoris*.

On the other hand, as represented by GS-XI-XK^SB50^, the cell growth was not improved while the xylose utilization rate was significantly increased (q_s_ was increased by 56%) after domestication (Figure 5D). As a result, the lowest Y_X/S_ on xylose (0.120 g/g) was obtained for GS-XI-XK^SB50^, which was only 32% of that of the GS-XI^SB50^ (Table 2). The results suggested that the increase in xylose utilization for GS-XI-XK^SB50^ was probably due to increased xylose dissimilation (such as respiration) in *P. pastoris*.

### Overexpression of XI and XK might be the key factors affecting the xylose assimilation

Due to their essential role in xylose conversion, XI and XK would conceivably undergo significant changes during evolution process. Therefore, examining the changes in expression levels of *XI* and *XK* might provide useful information on how improvements in xylose metabolic characteristics occurred and thus help guide further rounds of strain engineering.

**Figure 6:**
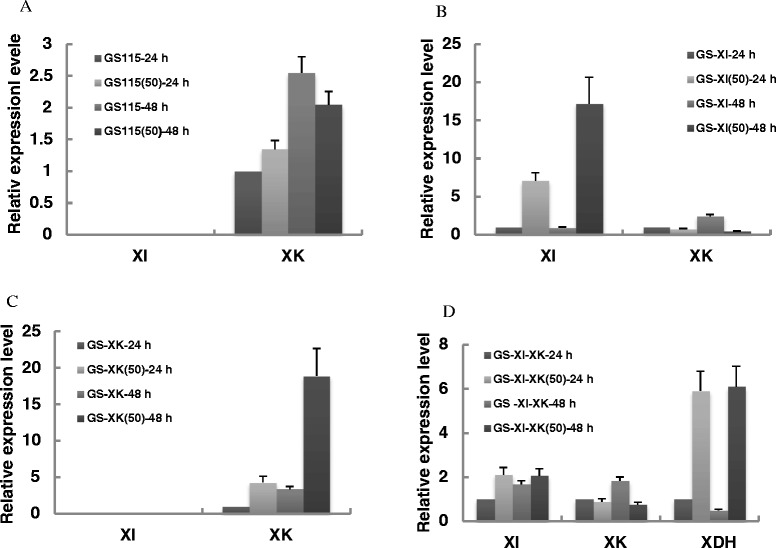
**Transcriptional changes of** ***XI***, ***XK*** **and** ***XDH*** **gene in evolved recombinant** ***Pichia pastoris*** **strains. (A)** GS115^SB50^. **(B)** GS-XI^SB50^. **(C)** GS-XK^SB50^. **(D)** GS-XI-XK^SB50^. All RNA samples were isolated from 24 and 48 h fermentation cultures. All experiments were performed in triplicate.

The *XI* and *XK* transcription levels in all four evolved strains together with their respective parental controls, were investigated by real time qPCR (Figure 6). Results showed *XI* transcription was increased by 6 ~ 16 fold in GS-XI^SB50^ (Figure 6B) while *XK* transcription was increased by 3.28 ~ 4.58 fold in GS-XK^SB50^ (Figure 6C). The remarkable up-regulation of *XI* and *XK* genes during evolution suggested that although already driven by the strong GAPDH promoter in the parent strains, the expression levels of XI and XK still could be the potential bottlenecks for effective xylose assimilation. This is especially true for XI, because despite the fact that XI had already achieved a considerably high expression level (accounting more than 4.5% of total soluble protein; Figure 3C) in the parent GS-XI strain (which would afflict yeast cells with great metabolic burden by occupying limited cellular resources), its expression level increased significantly after evolution, which illustrated the essential role XI plays in xylose assimilation. This conclusion was also in good consistence with a similar work performed in *S. cerevisiae* [26], where the authors by a comprehensive inverse metabolic engineering approach, concluded that elevated *XI* expression level was responsible for the efficient xylose assimilation in evolved strain.

For GS-XI-XK^SB50^, the up-regulation of *XI* or *XK* as seen in GS-XK^SB50^ and GS-XK^SB50^ were not observed (Figure 6D), which was quite unexpected because GS-XI-XK^SB50^ showed the highest xylose consumption rate among all strains. A possible explanation for this phenomenon was that the intrinsic oxidoreductase xylose pathway of *P. pastoris* strain GS-XI-XK rather than the constructed XI pathway evolved during the sequential batch cultivation. This may also explain the observed low cell yield and increased dissimilation phenotypes for GS-XI-XK^SB50^ because The oxidoreductase xylose pathway can result in redox imbalance and thus interfere with normal cellular physiology [29]. In order to test this possibility, the transcription of XR and XDH were also determined using real-time qPCR. Results showed 4.9 ~ 11.1 fold increase in XDH mRNA levels in GS-XI-XK^SB50^ (Figure 6D) while no significant difference in XR or XDH expression could be seen in other three evolved strains (data not shown). Therefore, increased XDH expression level could be responsible for increased xylose consumption rate in GS-XI-XK^SB50^, which was consistent with a previous study by Kim and his colleagues [30], where they observed that high level expression of XDH significantly increased xylose consumption rate (but not biomass yield) in *S. cerevisiae*.

In order to further investigate whether significant up-regulation of xylose pathway genes in evolved strains was due to multi-copy integration events during evolution as described in evolved recombinant *S. cerevisiae* [26], the relative copy number changes of GS-XK^SB50^/GS-XK, GS-XI^SB50^/GS-XI and GS-XI-XK^SB50^/GS-XI-XK were determined using qPCR. Results showed a six-fold change in *XI* copy number in GS-XI^SB50^ (Table 3). The enhancement of expression vector copy number in a single transformed cell line achieved by repeatedly plating it on high levels of a selectable marker drug, referred as post-transformational vector amplification (PTVA), has been previously described for *P. pastoris* [31], although the molecular mechanism is still unknown. This work demonstrated that in addition to drug marker system, PTVA also applied to other evolution pressure. In contrast, the up-regulation of XK in GS-XK and XDH in GS-XI-XK were not due to copy number increase (Table 3), which suggested more complex mechanisms might be involved in xylose pathway regulation. For a comprehensive examination of genetic factors leading to the improved xylose assimilation phenotype, systems biotechnology tools such as comparative genomics, transcriptomics analysis need to be further applied.

**Table 3:** **Changes of copy numbers of xylose pathway genes after evolutionary engineering**

**Strains**	**XI**	**XK**	**XDH**
GS-XI^SB50^/GS-XI	6.17 ± 0.76	N.D	N.D
GS-XK^SB50^/GS-XK	—	0.84 ± 0.27	N.D
GS-XI-XK^SB50^/GS-XI-XK	1.27 ± 0.07	N.D	1.03 ± 0.19

Copy number was determined by real time qPCR. *ACT1* gene was utilized as a reference control. Each sample was run in triplicates.

N.D: not determined, because no significant transcriptional changes were observed.

Therefore, the overexpression of XI and XK may be important to obtain *P. pastoris* strains with high efficiency of xylose assimilation in further strain improvement. Since the XI has already reached an extremely high expression level, further work to find alternative XIs with higher catalytic efficiency such as proposed by Lee and his colleagues [32] was highly desirable.

### β-Mannanase expression was improved in xylose utilizing P. pastoris in xylose medium

**Figure 7:**
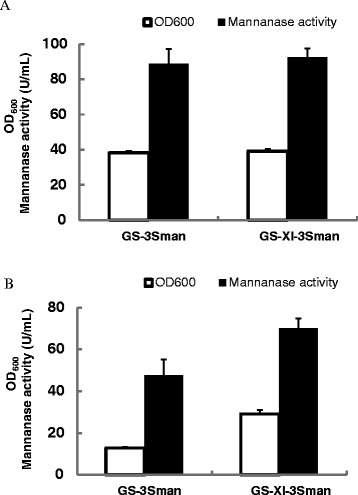
**Fermentation profiles of GS-3Sman and GS-XI-3Sman cultured on BMGY and BMXY media. (A)** Fermentation profiles of GS-3Sman and GS-XI-3Sman cultured on BMGY medium. **(B)** Fermentation profiles of GS-3Sman and GS-XI-3Sman cultured on BMXY medium.

To investigate whether xylose utilizing phenotype would result in enhanced heterologous protein expression on xylose medium as our final aim, an alkaline β-mannanase [20] was taken as an example to evaluate the protein expression traits of GS-XI^SB50^. The expression plasmid pGAPKH-3Sman was transformed into GS-XI^SB50^ and GS115 (used as a control), resulting in two recombinant strains GS-XI-3Sman and GS-3Sman, respectively. The two strains were first cultured with the BMGY medium in shaking-flasks. Results showed that two strains exhibited similar final cell biomass and β-mannanase production levels (Figure 7A), indicating that 50 generation of evolution did not affect the cell growth and enzyme expression profiles of engineered *P. pastoris* in glucose medium. When the two strains were cultured with xylose medium (BMXY), the final biomass of GS-XI-3Sman is significantly higher than that of GS-3Sman (126% increase) as expected. The final enzyme production of GS-XI-3Sman was 57.5% higher that of GS-3Sman (Figure 7B), showing that xylose utilizing *P. pastoris* is more productive in enzyme production (mainly due to increased cell growth) when cultured on xylose medium.

## Conclusions

For the first time, we demonstrated the successful engineering of xylose metabolizing ability into *P. pastoris* for industrial enzyme production. A heterologous XI pathway was introduced into *P. pastoris* and evolutionary engineering strategy was also applied. A recombinant *P. pastoris* strain was finally obtained with the highest xylose assimilation ability, whose cell yield on xylose was nearly two-fold higher than that of the starting strain GS115. This strain also showed significantly increased β-mannanase production when cultured on xylose medium. This work provided a basis for construction of cell factories with the potential to cost-effectively produce bulk enzymes from cellulosic biomass.

## Methods

### Strains and plasmids

All the plasmids and the strains used in this work are listed in Table 1, and the primers are concluded in Table 4. *Escherichia coli* (*E.coli*) DH 5α and *P. pastoris* GS115 were routinely used for vector construction. All primers were synthesized by Invitrogen (Beijing, China).

**Table 4:** **Primers used in this study**

**Primer name**	**Primer sequences (5′-3′)**	**Size (bp)**
5′ GAP	gtccctatttcaatcaattgaa	22
3′ AOX	gcaaatggcattctgacatcc	21
XI-F	atcaagaattcatgactaagga	22
XI-R	aaagctggcggccgcttactgat	23
XK-F	atcg*gaattc*atggttaccaaagaaatccaaa	32
XK-R	attg*gcggccgc*aaacgcctgacttgcttcac	32
rACT1-F	agtgttcccatcggtcgtag	20
rACT1-R	ggtgtggtgccagatctttt	20
rXI-F	catacgttatgcgctgatgg	20
rXI-R	ccctcgctcactagatcgac	20
rXK-F	tcttcatggcaaggaggaac	20
rXK-R	atcgaagacggcatgatagg	20
rXR-F	taccatcaccctgacaacgt	20
rXR-R	atccaccaacctctctagcg	20
rXDH-F	cccgtctcgttacagcaatg	20
rXDH-R	gcatggacagcaacactcaa	20

Enzyme sites were in italics.

### Growth and maintenance conditions

*E. coli* strains were cultivated aerobically at 37°C in Luria-Bertani medium (10 g/L NaCl, 10 g/L Tryptone (Thermo Fisher Oxoid, England) and 5 g/L Yeast Extract (Thermo Fisher Oxoid, England), pH 7.4 ~ 7.6) containing 25 mg/L Zeocin when required. All *P. pastoris* strains were grown at 30°C in YPD medium (20 g/L Glucose, 20 g/L Peptone (Becton, Dickinson and Company, America) and 10 g/L Yeast Extract) supplemented, when necessary, with 40 mg/L Zeocin for screening recombinant strains, while the HIS4^−^ recombinant *P. pastoris* strains were selected with MD solid medium (13.4 g/L YNB (Yeast Nitrogen Base w/o Amino Acid, Becton, Dickinson and Company, America), 0.4 mg/L Biotin, 20 g/L Glucose and 1.5% Agar). All *E. coli* and *P. pastoris* strains were maintained frozen in 25% glycerol at −80°C. Complex culture media used in this work were BMXY (8.7 g/L monopotassium phosphate, 13.4 g/L YNB, 0.4 mg/L Biotin, 20 g/L Peptone, 10 g/L Yeast Extract and 20 g/L Xylose, pH 6.0) and BMGY (identical to the BMXY except for the replacement of 20 g/L Xylose with 20 g/L glucose).

### Construction of recombinant plasmids

The *XI* gene from *Orpinomyces* spp. was chemically synthesized in Genewiz, Inc. (Suzhou, China) with codon optimized. This fragment was treated with *Eco*RI and *Not*I and inserted into the intracellular expression vector pGAPZH, thus generating the recombinant plasmid pGAPZ-XI-His (Figure 3A). The endogenous *XK* gene was cloned from genomic DNA of *P. pastoris* GS115 using XK-F and XK-R, creating *Eco*RI and *Not*I sites (italics in Table 4) and ligated to pGAPZmazF (E,N) to create the recombinant plasmids pGAPZ-XK (Figure 3B). The construction of the expression plasmid pGAPKH-3Sman, harboring three copies of alkaline β-mannanase gene was described in our previous work [33].

### Generation of recombinant *P. pastoris* strains

The recombinant plasmid pGAPZ-XK was linearized by *Avr*II and transformed into *P. pastoris* GS115 and screened on the YPD agar plate with Zeocin according to the protocol of Invitrogen, and the resulting positive transformants verified by colony PCR were designated GS-XK. The recombinant plasmid pGAPZ-XI-His was linearized by *Bsp*EI and separately transformed into GS115 and GS-XK and screened on the MD agar plate, thus generating the recombinant *P. pastoris* strain GS-XI and GS-XI-XK, respectively.

The expression plasmid pGAPKH-3Sman was linearized by *Bsp*EI and transformed into GS-XI^SB50^ and GS115, resulting in two recombinant strains GS-XI-3Sman and GS-3Sman, respectively.

### Shaking-flask fermentation

Shaking-flask fermentation of the recombinant strains GS-XI, GS-XK and GS-XI-XK were performed with the GS115 as the control. All strains were first grown in YPD medium and then transferred into the BMXY medium with an initial OD_600_ of around 8. All cultures were performed at 30°C, 200 r/m, for 120 h. The cell concentration was determined from the OD_600_ value. Xylose concentration was analyzed by HPLC (HP1260; Agilent) using an Aminex HPX-87H ion-exchange column (7.8 × 300 mm). The mobile phase was 0.05 mmol/L sulfuric acid and the flow rate was 0.5 mL/min. The recombinant strain GS-XI-3Sman was cultured in the BMGY or BMXY at 30°C and 200 r/m for 96 h, respectively, and the strain GS-3Sman used as a control.

### Evolutionary engineering of recombinant *P. pastoris*

Four strains GS115, GS-XI, GS-XK and GS-XI-XK were used to initiate the evolution process, which was performed by sequential batch cultivation under aerobic conditions in BMXY medium at 30°C with 200 r/m. When the culture reached the stationary phase (4 ~ 6 d), an aliquot (1 mL) was transferred into a fresh 100 mL flask containing 25 mL BMXY liquid medium to start a new batch. The whole evolution process lasted for 50 generations (approximately one year), and the evolved strains were referred as GS-XI^SB50^, GS-XK^SB50^, GS-XI-XK^SB50^ and GS115^SB50^, correspondingly.

### Genomic DNA preparation, RNA extraction and cDNA synthesis

Genomic DNA was prepared by Bust n’ Grab method [34]. Total RNA of each sample was prepared using an RNAprep pure kit (Tiangen Biotech, Co., Ltd, Beijing, China) following the manufacturer’s recommendations. RNA samples were stored at −80°C until used. 2 μg of each total RNA was subjected to reverse transcription using the Fast Quant RT Kit (Tiangen Biotech, Co., Ltd, Beijing, China).

### Real-time qPCR for transcriptional analysis and copy number determination

The PCR primer design was conducted using Primer3 software (http://primer3.ut.ee/). All the primers used in this work were listed in Table 4. The reaction conditions had been established as recommended by SYBR®*Premix ExTaq*™ manual (TaKaRa Bio Co., Ltd, Dalian, China). Each 20 μL reaction contained 10 μL 2× SYBR Premix Ex Taq™, 0.4 μL 50 μM forward and reverse primers, 2.0 μL sample cDNA, and 7.2 μL nuclease-free water. All real-time qPCR reactions were run in triplicate on a Light Cycler® 96 (Roche, Switzerland) using the following program: 95°C for 3 min, 45 cycles of 95°C for 5 s, and 60°C for 20 s. The specificity of amplicons were verified by melting curve analysis after 40 cycles and agarose gel electrophoresis. The 2^-ΔΔ^ method was used for analyze *XI*, *XK*, *XR* and *XDH* gene in different strains with *ACT1* gene as the endogenous control (housekeeping gene). The copy numbers of genes *XI*, *XK* and *XDH* were determined following methods described previously [35].

### Protein analysis and enzyme activity determination

Protein concentrations were determined using the Bradford method with bovine serum albumin (BSA) as a standard. Sodium dodecyl sulfate-polyacrylamide gel electrophoresis (SDS-PAGE) was performed on a 10% polyacrylamide gel with precision protein marker. To identify the target protein, protein bands in gel were excised and subjected to in-gel-digestion and matrix-assisted laser desorption ionization–time of flight mass spectrometry (MALDI-TOF MS) analysis. XI expression was estimated by optical density scanning using Gel-Pro Analyzer software (Media Cybemetics).

β-Mannanase activity was determined as described by Ma et al. [36]. One unit of β-mannanase activity was defined as the amount of enzyme that liberated 1 μmol of reducing sugar per minute with locust bean gum as substrate.
